# The impact of *ERBB2* alterations in muscle‐invasive bladder cancer after neoadjuvant chemotherapy

**DOI:** 10.1002/bco2.70274

**Published:** 2026-08-16

**Authors:** Earle F. Burgess, Hilary Elom, Landon C. Brown, Michael McCormack, Ericson Stoen, Metamia Ciampricotti, Edward Williams, James T. Symanowski

**Affiliations:** ^1^ Atrium Health Wake Forest Baptist Comprehensive Cancer Center Charlotte North Carolina USA; ^2^ Atrium Health Wake Forest Baptist Comprehensive Cancer Center Winston‐Salem North Carolina USA; ^3^ Tempus AI, Inc. Chicago Illinois USA

**Keywords:** biomarker, bladder cancer, *ERBB2*, HER2, neoadjuvant chemotherapy, urothelial carcinoma

## 1 INTRODUCTION

Cisplatin‐based neoadjuvant chemotherapy (NAC) has been a standard treatment for muscle‐invasive bladder cancer (MIBC), although peri‐operative management is evolving to include immune checkpoint inhibitor (ICI) and enfortumab vedotin (EV)‐based regimens.^1^, ^2^ Despite these advances, access to EV and ICIs remains limited in much of the world, and cisplatin‐based NAC continues to represent a standard option for a large proportion of patients globally. Identification of biomarkers associated with response to NAC therefore remains clinically important.

Both pathogenic *ERBB2* mutations and gene amplification are well‐recognized genomic alterations in MIBC.^3^ Prior studies have reported conflicting findings regarding the association of *ERBB2* mutations and amplification with response to NAC in MIBC,^4^, ^5^, ^6^ which may be due to heterogeneity in chemotherapy regimens, genomic sequencing methodologies and definitions of treatment response across these studies. These discordant results highlight the need for additional study to clarify the prognostic value of *ERBB2* alterations in MIBC.

We previously reported that tumour mutational burden (TMB) ≥ 10 mutations per megabase (Mut/Mb) was associated with improvement in pathologic complete response rate (pCR), relapse‐free survival (RFS) and overall survival (OS) in a retrospective cohort of 91 patients with MIBC treated with cisplatin‐based NAC followed by radical cystectomy.^7^ In this cohort, all patients received NAC with either gemcitabine and cisplatin or standard or dose‐dense methotrexate, vinblastine, doxorubicin and cisplatin (MVAC) across two campuses at our institution. Only four (4.4%) patients received adjuvant nivolumab, minimizing the potential for ICI‐related confounding of survival outcomes in this cohort. Genomic profiling of pretreatment transurethral resection specimens was performed using the commercially available Tempus xT next‐generation sequencing (NGS) platform. Associations between *ERBB2* gene variants, TMB and clinical outcomes were assessed using logistic regression, Cox proportional hazards models and Kaplan–Meier techniques.

*ERBB2* alterations were identified in 17/91 (18.7%) patients, including four pathogenic mutations (4.4%) and 13 amplifications (14.3%); all mutations were p.S310F. pCR was achieved in 29/91 (31.9%) patients overall. Among those with *ERBB2* alterations, pCR occurred in 3/4 (75.0%) patients with a mutation and 6/13 (46.2%) patients with amplification. In aggregate, the presence of an *ERBB2* alteration (mutation + amplification) was associated with improved likelihood of pCR compared to non‐*ERBB2* altered patients (OR 3.038, *p* = 0.048).

**FIGURE 1:**
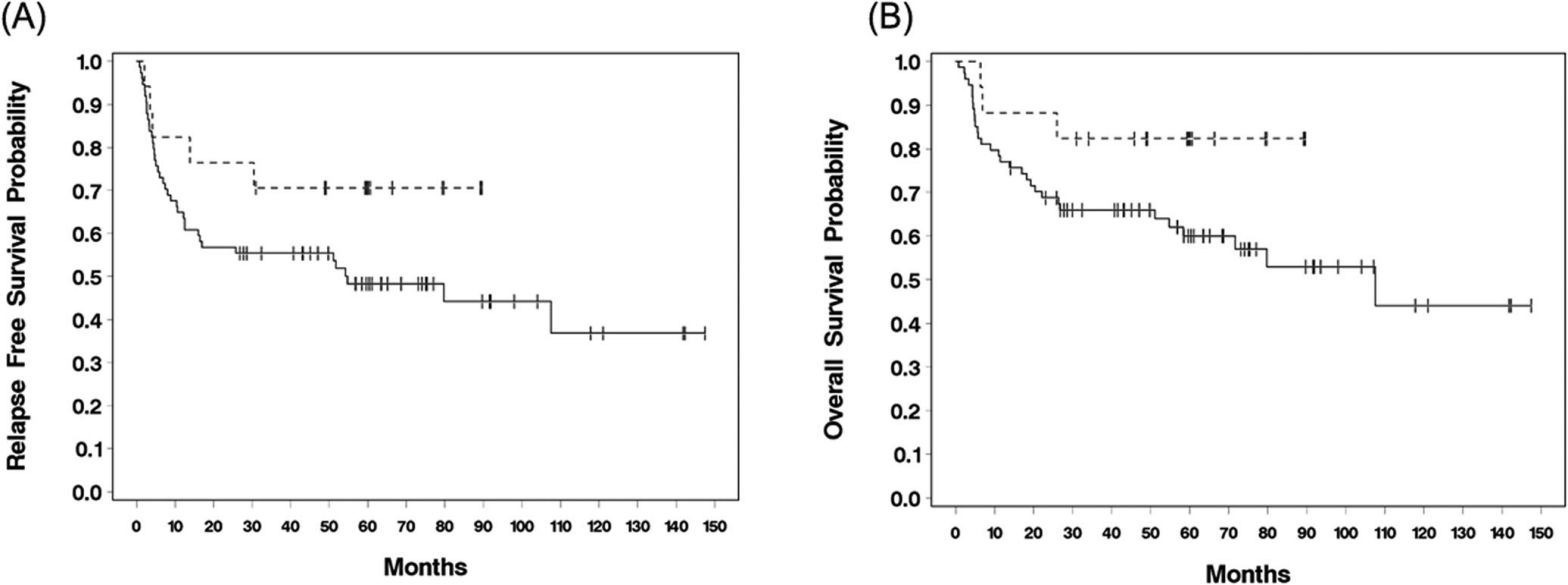
Relapse‐free survival (A) and overall survival (B) by ERBB2 status. ERBB2 Mut + amp (dotted line, N = 17). ERBB2 wild type (solid line, N = 74).

Given the favourable impact of *ERBB2* alterations on pCR, we next assessed survival endpoints. Median follow‐up for the entire cohort was 63.6 months; 38/91 (41.8%) relapsed. Five of 17 (29.4%) patients with an *ERBB2* alteration relapsed. Median RFS was not reached in the *ERBB2*‐altered group versus 54.7 months in the wild‐type group (HR 0.50, *p* = 0.134; Figure 1A). Median OS was not reached versus 107.6 months, respectively (HR 0.381, *p* = 0.098; Figure 1B). Although we previously reported that TMB ≥ 10 Mut/Mb was associated with improved pCR, RFS and OS in this cohort, the presence of an *ERBB2* alteration was not clearly associated with TMB ≥ 10 Mut/Mb (OR 2.217, *p* = 0.178).

To our knowledge, this is the first study to report the clinical impact of *ERBB2* gene alterations detected by a widely available, contemporary NGS platform in a uniformly treated cohort of MIBC patients receiving cisplatin‐based NAC. Although this study was not powered to detect definitive associations with survival endpoints, concordant trends towards improved RFS and OS were observed in patients with *ERBB2* alterations. The lack of a clear association between *ERBB2* alteration and high TMB further suggests that *ERBB2*‐driven chemosensitivity may operate through mechanisms distinct from those associated with high TMB.

These findings should be interpreted in the context of several limitations, including modest sample size, retrospective design and a single‐institution cohort. However, this cohort is among the largest reported to assess the prognostic value of ERBB2 alterations in NAC‐treated MIBC, benefits from mature follow‐up, uniform cisplatin‐based NAC administration and a rigorous definition of pathologic response (ypT0ypN0). While applicability may be limited in regions where treatment paradigms have shifted to include peri‐operative ICIs or EV‐based regimens, these approaches remain inaccessible to a substantial proportion of patients globally, and cisplatin‐based NAC is expected to remain standard of care in many settings. These findings also raise the question of whether ERBB2 alteration retains prognostic relevance in patients treated with contemporary peri‐operative ICI and EV‐based regimens, which warrants prospective investigation.

In summary, *ERBB2* alterations detected by a routinely available NGS platform were associated with improved pCR and concordant trends towards improved RFS and OS in a uniformly treated cisplatin‐based NAC cohort. These findings support the continued need to identify prognostic biomarkers in MIBC, particularly for the large proportion of patients globally who lack access to contemporary peri‐operative regimens.

## AUTHOR CONTRIBUTIONS

Conceptualization: EFB, JTS. Methodology: EFB, LCB, MM, ES, JTS. Data Analysis: EFB, LCB, MM, ES, JTS. Writing—original draft: EFB, JTS. Writing—review and editing: All authors. Funding acquisition: EFB.

## CONFLICT OF INTEREST STATEMENT

M. Ciampricotti and E. Williams are employees of Tempus AI, Inc. The authors declare no other relevant conflict of interests.

## Data Availability

The data that support the findings of this study are available from the corresponding author upon reasonable request.

## References

[1] Vulsteke C , Adra N , Danchaivijitr P , Sabadash M , Rodriguez‐Vida A , Zhang Z , et al. Perioperative enfortumab vedotin and pembrolizumab in bladder cancer. N Engl J Med. 2026;394(13):1257–1269. 10.1056/NEJMoa2511674 41707170

[2] Powles T , Catto JWF , Galsky MD , al‐Ahmadie H , Meeks JJ , Nishiyama H , et al. Perioperative durvalumab with neoadjuvant chemotherapy in operable bladder cancer. N Engl J Med. 2024;391(19):1773–1786. 10.1056/NEJMoa2408154 39282910

[3] Robertson AG , Kim J , Al‐Ahmadie H , Bellmunt J , Guo G , Cherniack AD , et al. Comprehensive molecular characterization of muscle‐invasive bladder cancer. Cell. 2017;171(3):540–556.e25. 10.1016/j.cell.2017.09.007 28988769 PMC5687509

[4] Groenendijk FH , de Jong J , Fransen van de Putte EE , Michaut M , Schlicker A , Peters D , et al. *ERBB2* mutations characterize a subgroup of muscle‐invasive bladder cancers with excellent response to neoadjuvant chemotherapy. Eur Urol. 2016;69(3):384–388. 10.1016/j.eururo.2015.01.014 25636205

[5] Gil‐Jimenez A , van Dorp J , Contreras‐Sanz A , van der Vos K , Vis DJ , Braaf L , et al. Assessment of predictive genomic biomarkers for response to cisplatin‐based neoadjuvant chemotherapy in bladder cancer. Eur Urol. 2023;83(4):313–317. 10.1016/j.eururo.2022.07.023 35965206

[6] Kiss B , Wyatt AW , Douglas J , Skuginna V , Mo F , Anderson S , et al. Her2 alterations in muscle‐invasive bladder cancer: patient selection beyond protein expression for targeted therapy. Sci Rep. 2017;7(1):42713. 10.1038/srep42713 28205537 PMC5311866

[7] Burgess EF , Brown LC , McCormack MJ , Stoen E , Doherty P , Williams E , et al. Impact of tumor mutational burden on clinical outcomes in muscle‐invasive bladder cancer patients treated with neoadjuvant chemotherapy. J Clin Oncol. 2026;44(7_suppl):814–814. 10.1200/JCO.2026.44.7_suppl.814 PMC13519591 42594312

